# Chloroplastic Hsp100 chaperones ClpC2 and ClpD interact *in vitro* with a transit peptide only when it is located at the N-terminus of a protein

**DOI:** 10.1186/1471-2229-12-57

**Published:** 2012-04-30

**Authors:** Eduardo M Bruch, Germán L Rosano, Eduardo A Ceccarelli

**Affiliations:** 1 Molecular Biology Division, Instituto de Biología Molecular y Celular de Rosario (IBR), CONICET, Facultad de Ciencias Bioquímicas y Farmacéuticas, Universidad Nacional de Rosario, Suipacha 531, S2002LRK, Rosario, Argentina

**Keywords:** Hsp100 chaperones, Clp, Chloroplasts, Protein import, Transit peptide

## Abstract

**Background:**

Clp/Hsp100 chaperones are involved in protein quality control. They act as independent units or in conjunction with a proteolytic core to degrade irreversibly damaged proteins. Clp chaperones from plant chloroplasts have been also implicated in the process of precursor import, along with Hsp70 chaperones. They are thought to pull the precursors in as the transit peptides enter the organelle. How Clp chaperones identify their substrates and engage in their processing is not known. This information may lie in the position, sequence or structure of the Clp recognition motifs.

**Results:**

We tested the influence of the position of the transit peptide on the interaction with two chloroplastic Clp chaperones, ClpC2 and ClpD from *Arabidopsis thaliana* (AtClpC2 and AtClpD). The transit peptide of ferredoxin-NADP^+^ reductase was fused to either the N- or C-terminal end of glutathione *S*-transferase. Another fusion with the transit peptide interleaved between two folded proteins was used to probe if AtClpC2 and AtClpD could recognize tags located in the interior of a polypeptide. We also used a mutated transit peptide that is not targeted by Hsp70 chaperones (TP1234), yet it is imported at a normal rate. The fusions were immobilized on resins and the purified recombinant chaperones were added. After a washing protocol, the amount of bound chaperone was assessed. Both AtClpC2 and AtClpD interacted with the transit peptides when they were located at the N-terminal position of a protein, but not when they were allocated to the C-terminal end or at the interior of a polypeptide.

**Conclusions:**

AtClpC2 and AtClpD have a positional preference for interacting with a transit peptide. In particular, the localization of the signal sequence at the N-terminal end of a protein seems mandatory for interaction to take place. Our results have implications for the understanding of protein quality control and precursor import in chloroplasts.

## Background

Clp/Hsp100 proteins are a class of hexameric molecular chaperones from the ever-growing family of AAA + (*A*TPases *a*ssociated with diverse cellular *a*ctivities) ATPases. They possess a wide range of functional roles, including protein folding assistance, protein degradation, disaggregation of denatured polypeptides and assembly of large molecular complexes [1-3]. Most (if not all) Clp/Hsp100 proteins act as independent chaperones but some associate with oligomeric barrel-shaped proteases to degrade irreversibly damaged proteins [4,5]. Notable examples are ClpA and ClpX from *Escherichia coli* and ClpC from *Bacillus subtilis* and *Synechococcus elongatus*, which couple to their respective ClpP protease [6-8].

In plants, the diversity of the Clp family is greater than in any other organism. The model plant *A. thaliana* has at least 23 members [9]. Hsp100 chaperones from the ClpX family (ClpX1-3) are found in mitochondria while those from the ClpC (ClpC1 and ClpC2) and ClpD subtype are chloroplastic. The ClpPR proteolytic core is represented by six different proteins (ClpP1-6) and four ClpR proteins (ClpR1-4). Three modulator/adaptor proteins (ClpT1, ClpT2 and ClpS) are thought to regulate substrate recognition and binding.

Recombinant AtClpC2 and AtClpD have been recently characterized [10]. AtClpD was consistently obtained as two forms, one being the full-length protein and the other corresponding to a C-terminus-processed variant. The proteins displayed ATPase and foldase activity and self-assembled into hexamers. Clp/Hsp100 chaperones are constitutively expressed and are highly conserved among different plant species [11]. Knockout plants in the *Atclpc1* gene are pale green and show retarded growth, while *Atclpc1*/*Atclpc2* double mutants are not viable [12]. All this evidence suggests that chloroplastic Clp proteins are key members in the process of protein quality control in the chloroplast.

In addition to their proposed housekeeping duties, ClpC proteins from plants have been implicated in the import of nuclear-encoded proteins into chloroplasts. Proteins destined to the organelle are synthesized as higher molecular mass precursors containing an N-terminal extension called transit peptide (TP). It acts as a signal that is recognized by the translocon channel, a bipartite complex with central pores through which precursors penetrate into the plastid stroma [13,14]. A fraction of ClpC is localized in the stromal side of the chloroplast inner membrane and interacts with the import machinery when a precursor is being translocated [10,15]. In *A. thaliana*, chloroplasts isolated from insertional mutants in the *clpc1* gene show a diminished rate of import [12,16,17]. The current model of protein import into chloroplasts place Hsp100 chaperones (specifically, members of the ClpC subfamily) as motors pulling precursors by their TPs. Yet many aspects of this scenario are still to be elucidated, such as TP recognition by the chaperone, threading of the polypeptide through the Hsp100 ring and interplay with the Hsp70 network, which was also shown to interact with precursors [18].

The step of substrate recognition has been studied in ClpA from *E. coli*. This chaperone can recognize tags located at the N- or C-terminal end (e.g., the RepA tag and the SsrA tag, respectively) [19,20]. Though the amino acid composition of these tags is well characterized, consensus sequences are still not defined, as many other untagged proteins are substrates of ClpA [21]. It is thought that short exposed segments with hydrophobic amino acids and little (if any) tridimensional structure are recognized, like is the case for the substrates of Lon protease and the chaperones GroEL, DnaK, Trigger Factor and many others [22]. However, it is not known which specific attributes are recognized by the Hsp100 chaperones associated with the translocation channel. Furthermore, since only a handful of Hsp100 targets have been identified, no obvious sequence pattern is available to analyze if a given TP could be an Hsp100 substrate.

The aim of this work was to elucidate some aspects of the process of TP recognition by Clp/Hsp100 chaperones. Several fusion proteins were constructed in which TPs were placed at different positions in a polypeptide. Then, binding to these probes by purified recombinant AtClpC2 and AtClpD was analyzed. Using this *in vitro* system, we found that AtClpC2 and AtClpD only interacted with the fusions that have the TPs in the natural location (i.e., at the N-terminus).

## Results and discussion

### AtClpC2 and AtClpD interact with transit peptides located in the N-terminal end

**Figure 1:**
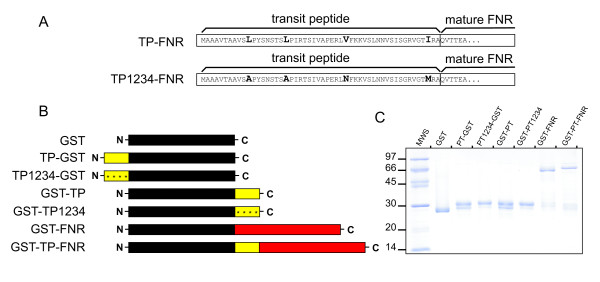
**Depiction of the fusion proteins used in this study.** (**A**) Amino acid sequence of wild type TP and TP1234. Substituted amino acids are shown in bold. (**B**) Proteins used throughout the study. Yellow boxes represent either TP (5 kDa), black boxes GST (27 kDa) and red boxes FNR (35 kDa). (**C**) Purity and integrity of GST and GST-fusions, checked by SDS-PAGE followed by Coomassie blue staining. Left lane: molecular weight standard.

As a recognition sequence for the chaperones, we chose the TP of pea ferredoxin-NADP^+^ reductase (FNR). This TP interacts with several stromal chaperones involved in protein import [10,23]. We also used a mutant form of the TP (TP1234) in which the Hsp70 recognition sites have been eliminated by site-directed mutagenesis [24]. The mutations cover the entire length of the TP and are arranged at regular intervals (Figure 1A). These TPs were fused in different configurations to glutathione *S*-transferase (GST, Figure 1B) in order to probe the effect of TP location on the interaction with the Clp chaperones. The GST-fusions were expressed in *E. coli* and bound to glutathione-agarose resins. To check their purity and integrity, they were analyzed by SDS-PAGE after elution from the resins. It can be seen in Figure 1C that the fusions were not subjected to extensive proteolytic cleavage and were devoid of contaminating proteins. However, it can be noted that TP-bearing fusions are recovered as a mixture of full length protein and intermediate variants, resulting from cleavage of the TP at different sites [10].

**Figure 2:**
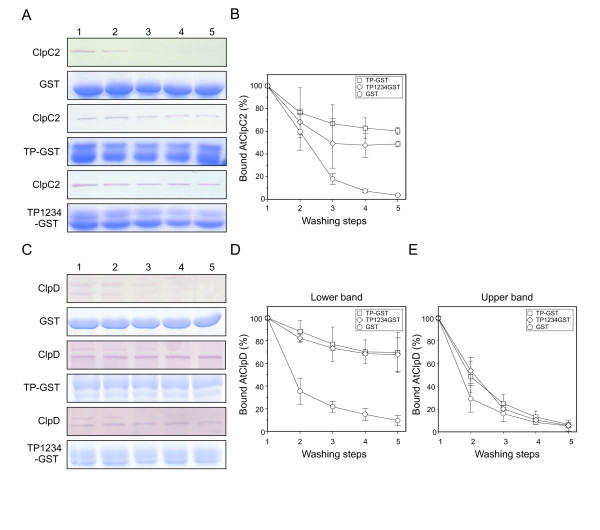
**Interaction of AtClpC2 and AtClpD with TPs located at the N-terminal end of GST.** GST, TP-GST or TP1234-GST were used to load the gluthathione-agarose resin. Blots show the remaining AtClpC2 (**A**) or AtClpD (**C**) after each wash. Coomassie blue-stained SDS-PAGE gels below each blot show the integrity of GST and GST fusion proteins along the washing steps. Digitized bands from six different blots were quantified using the software GelPro. Graphs show the percentage of residual AtClpC2 (**B**) or AtClpD (**D**, lower band; **E,** upper band) in the TP-GST- (squares), the TP1234-GST- (diamonds), or in the GST-containing resin (circles) after each washing step.

The fusion proteins containing the TPs at their N-terminal end were loaded onto a glutathione-agarose resin. Recombinant AtClpC2 or AtClpD were added to the fusion- or GST-containing resins and incubated for 30 minutes in the presence of 5 mM ATP. Then, the beads were subjected to several washing steps and the amount of remaining chaperone bound to the resin after each step was analyzed by SDS-PAGE and immunoblotting. The rationale of the experimental strategy is as following: if the chaperones stably interacted with the TPs then, after the washing protocol, they would still be bound to the TPs-containing resin. In contrast, Hsp100 chaperones loaded in the GST-containing resin should be completely washed away. Blots showing the amount of remaining AtClpC2 (Figure 2A) and AtClpD (Figure 2C) after each step is shown. Bands were quantified by densitometry and the percentage of bound chaperone for each step was plotted. The graph for AtClpC2 is shown in Figure 2B. Since AtClpD is expressed as two different forms, each was plotted separately (Figure 2D, lower band; Figure 2E, upper band). The amount of either chaperone bound to the TP-containing beads after five washing steps is more than 65% of the initial loaded amount. This was also the case for the TP1234-containing resin, although AtClpC2 showed a higher release for this variant, as less than 50% of the initial loaded amount could be detected after the washing steps. Full length AtClpD is completely lost during the procedure. To rule out that the chaperones were binding to the carrier GST, AtClpC2 and AtClpD were added to a GST-containing resin. No binding could be detected, which indicates that the interaction seen with the fusions was specific to the TP moiety. Chaperone removal from the resins was not due to degradation or loss of the fusions during the washing procedure, as fusion content after each step was checked by SDS-PAGE. Coomassie blue-stained gels are shown below each corresponding blot.

The interaction of AtClpC2 and AtClpD with a TP located at N-terminal end of a given protein was rather expected. This situation is present in protein translocation across membranes, where the N-termini of precursors enter the organelle and may be met by AtClpC1/2 (and possibly AtClpD) that pull them in. The fact that only the C-terminal processed variant of AtClpD interacted with the fusions suggests that this would be the active form, at least under these conditions. However, no evidence exists about chaperone activity regulation by C-terminal processing. This could represent a novel mechanism to adjust the quantity of active AtClpD.

We also used TP1234-GST in this study to investigate why TP1234-bearing proteins are efficiently imported to chloroplasts, considering that they are not targeted by Hsp70 chaperones [24]. Su *et al*. postulated that Hsp100 and Hsp70 chaperones associate to precursors independently rather than cooperatively [18]. They arrived at this conclusion after noting that *A. thaliana* double mutant plants in the dominant paralogs of Hsp70 and Hsp100 (cpHsc70-1 and ClpC1, respectively) showed additive import defects. The fact that the chaperones readily associated with TP1234-GST supports this model: since Hsp70 chaperones do not interact with TP1234, then AtClpC1 or AtClpC2 (and possibly AtClpD) provide the driving force for its import, without the aid of another chaperone system.

### AtClpC2 and AtClpD cannot recognize a transit peptide with blocked termini

**Figure 3:**
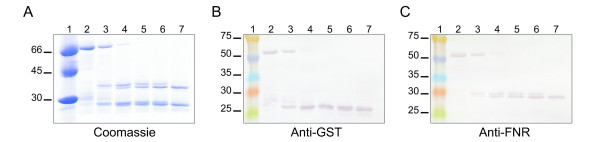
**Limited proteolysis assays. GST-TP-FNR was digested with thermolysin.** At different time points (see Materials and Methods), aliquots were taken and analyzed by SDS-PAGE stained with Coomassie Brilliant Blue (**A**), followed by immublotting revealed with antibodies raised against GST (**B**) or FNR (**C**). Lanes 1 show the migration of molecular weight standards.

We next tested if the chaperones could interact with the TP with its terminal ends blocked. A fusion protein where the TP was inserted between two proteins was used (GST-TP-FNR). In order for a Clp hexamer to be positioned between the two proteins, there should be enough space available. Crystallographic data indicate that the height of a ClpA hexamer is 87 Å [25], which should be taken as the minimal distance required for a hexamer to accommodate between GST and FNR. Although this data is not available for Clp/Hsp100 proteins from plants, it should be a good approximation. Analysis of the GST-TP-FNR construct indicates that GST and FNR are separated by a segment of 81 unstructured amino acids (Additional file 1: Table S1). The distance between two consecutive amino acids has been estimated to be 1.5 Å for an alpha helix, 2 to 2.5 Å for a random coil and 3.5 Å for a fully extended conformation [26,27]. This means that GST and FNR should be between 121 Å (full alpha helix) and 283 Å (fully extended conformation) apart. This is more than the required space of 87 Å. This calculation aside; it could be possible that TP would get buried between GST and FNR, rendering it inaccessible to the chaperones. Previous work by our laboratory using this fusion demonstrated that bacterial and plant Hsp70 interacted with the TP with blocked termini, indicating that it was accessible to the solvent [23]. To confirm this finding, limited proteolysis experiments were carried out. Purified GST-TP-FNR was incubated in the presence of the protease thermolysin. After different time points, aliquots were taken and analyzed by SDS-PAGE and immunoblotting (Figure 3). In all cases, two bands corresponding to FNR and GST were clearly resolved. This result indicates that the protease had access to the TP linking both proteins.

**Figure 4:**
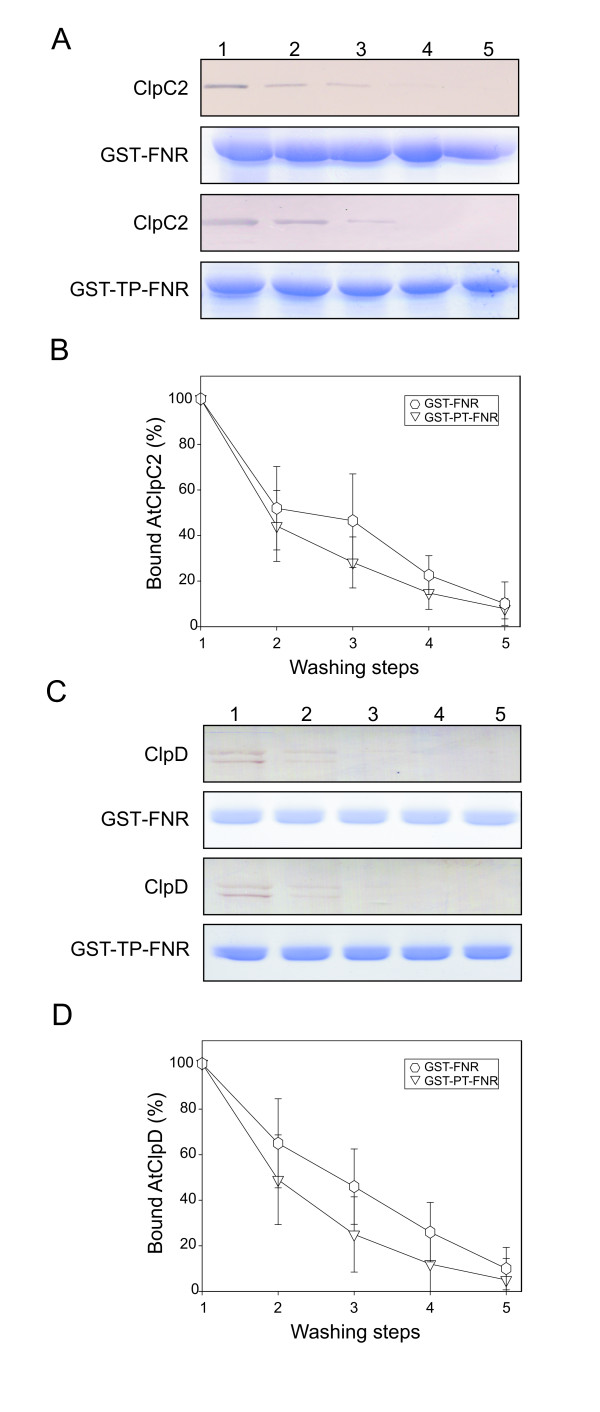
**Binding of AtClpC2 and AtClpD to the wild-type TP located between GST and FNR.** GST-FNR or GST-TP-FNR were used to load the gluthathione-agarose resin. Representative blots from three different experiments show the remaining AtClpC2 (**A**) or AtClpD (**C**) after each washing step. Graphs show the percentage of residual AtClpC2 (**B**) or AtClpD (**D**) in the GST-FNR- (hexagons) or GST-TP-FNR-containing resin (inverted triangles) after each washing step. For AtClpD, only the lower band was plotted, as identical results were obtained with the upper band. Coomassie blue-stained SDS-PAGE gels below each blot show the integrity of GST fusion proteins along the washing steps.

Next, the GST-TP-FNR fusion was expressed and used to load the glutathione-agarose resin. Then, the same experimental steps described in the preceding section were carried out. As a control, a GST-FNR fusion (without any linker) was used. In this case, both AtClpC2 and AtClpD were washed from the GST-TP-FNR loaded resin in the same way as the control GST-FNR, as judged by the blots shown in Figure 4A and C, respectively. Quantification of band intensity for each blot is plotted in Figure 4C and D. For AtClpD, only the lower band was plotted (the upper band behaved in a similar way). These results show that the chaperones did not interact with the TP when its terminal ends were blocked.

For internally tagged substrates, AtClpC2 and AtClpD chaperones behave as their bacterial counterparts, i.e., they do not recognize the tags when located between two proteins [28]. This lack of recognition rules out two probable scenarios. First, it could be possible that a loop containing the recognition sequence is targeted by the chaperones, since the central pore present in Clp hexamers is wide enough to accommodate two polypeptide chains simultaneously [29]. In this case, the presence of a free end would not be necessary. A second possibility would be that dimers of the Clp chaperones continuously scan for targets and upon finding them, the hexamer would form over the sequence. This situation would also avoid the requirement for a free end. Yet, our results and those of others do not support these models, at least *in vitro*. The situation *in vivo* may be somewhat different. Other proteins may aid in substrate presentation or in the assembly and disassembly of the hexamers, making the above hypotheses still plausible.

### AtClpC2 and AtClpD cannot recognize transit peptides located at the C-terminal end of a protein

**Figure 5:**
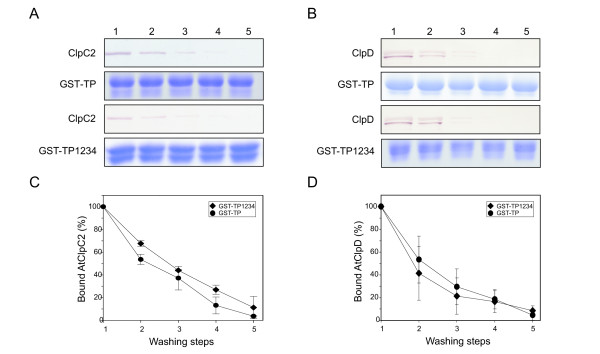
**Interaction of AtClpC2 and AtClpD with TPs located at the C-terminal end of GST.** GST-TP or GST-TP1234 were loaded onto the glutathione-agarose resin. Blots show the remaining AtClpC2 (**A**) or AtClpD (**B**) after each washing step. Coomassie blue-stained SDS-PAGE gels below each blot show the integrity of GST fusion proteins along the procedure. Graphs indicate the percentage of residual AtClpC2 (**C**) or AtClpD (**D**) in the GST-TP (filled circles) or in the GST-TP1234-containing resin (filled diamonds) after each washing step. For AtClpD, only the lower band was plotted, as identical results were obtained with the upper band. Results from three different experiments are shown.

The N-terminal region of the ClpA-substrate RepA can be transferred to either end of a polypeptide and direct it to degradation. This observation demonstrated that this tag is an autonomous entity [30]. To test if this was also the case for AtClpC2 and AtClpD and the TP of FNR, two fusion proteins were constructed where TP and TP1234 were placed at the C-terminal end of GST. Glutathione-agarose resins were loaded with the fusion proteins and interaction with AtClpC2 or AtClpD was analyzed as before. The results are presented in Figure 5. It can be seen that both AtClpC2 and AtClpD were washed from the TPs-containing resins, indicating that interaction with the TPs did not take place.

Taken together, our results show that tag position seems to be important for substrate recognition by Clp chaperones from plants, as observed for the TP of pea FNR. By contrast, bacterial Clp chaperones such as ClpA and ClpX from *E. coli* do not discriminate against the position of the sequence tag [28,31]. For example, the first 15 amino acids of the ClpA-substrate RepA are sufficient to tag any protein for degradation by ClpP. When this ClpA-recognition motif is allocated to the C-terminal end, efficient degradation by ClpAP ensues. Moreover, when the RepA tag was placed at the C-terminal end of the green fluorescent protein but in an inverse orientation, the tagged protein was still unfolded by ClpA as judged by a loss in fluorescence. Other experiments were carried out using a ClpX-specific tag (the last ten amino acids of its substrate MuA) with similar results. These reports from the Wickner lab showed that bacterial Clp chaperones promote substrate unfolding, regardless of the position and orientation of the tag. Why this different mode of action between plant and bacterial chaperones exists is not clear. Precursors may represent a subset of Clp/Hsp100 substrates with particular recognition rules. More work is needed to fully understand how stromal chaperones bind to precursors and other targets.

## Conclusions

In this work, we show that AtClpC2 and AtClpD from *A. thaliana* interact with the transit peptide of pea FNR located at the N-terminal end of a polypeptide. Its relocation to the C-terminal end or to the interior of a protein abolishes the interaction. The chaperones could also bind to a mutated TP not recognizable by Hsp70 chaperones, explaining why fusion proteins bearing this TP are efficiently imported into chloroplasts. Altogether, our results agree with the role of Clp/Hsp100 chaperones in the binding and pulling of incoming proteins into the chloroplastic stroma.

## Methods

### Plasmid construction

The DNA coding sequence of TP and TP1234 of pea FNR were amplified by PCR from the plasmids pGF202b and pGF202b-1234 [23]*.* The primers contained an NcoI and a SacI restriction sites that allowed directional cloning into a modified pET32 vector called pETGEXCT [32], resulting in plasmids pETGEX-TP and pETGEX-TP1234. These expression vectors produce each TP fused to the N-terminal end of GST. Construction of plasmids pGF202 and pGF205, which express GST fused to the N-terminal end of the FNR precursor (fusion GST-TP-FNR) or mature FNR (fusion GST-FNR), respectively, has been described elsewhere [33,34]. Plasmids pDR52 and pDR52b [23] encode the proteins GST-TP and GST-TP1234, respectively, where the TPs are fused to the C-terminal region of GST. All constructs used in this study were checked by DNA sequencing.

### Protein expression and purification

The plasmids were used to transform the *E. coli* strain BL21(DE3). Transformed cells were grown in Luria-Bertani medium containing the corresponding antibiotics at 37°C until the suspension reached an optical density of 0.7 at 600 nm. Afterwards, the cells were cooled to 18°C, induced by adding 0.5 mM isopropyl-β-D-thiogalactopyranoside and grown for 16 hours at 18°C. Cells were collected by centrifugation, resuspended in cold lysis buffer (50 mM Tris–HCl, pH 8.0, 150 mM NaCl, 1 mM phenylmethylsulfonyl fluoride, 1 mM benzamidine, 1 mM dithiothreitol, 5 mM EDTA, 10% v/v glycerol) in a 20:1 ratio (culture:lysis buffer) and sonicated in an ice-water bath. All subsequent steps were performed at 4°C. The soluble fraction was obtained by centrifugation (30000 × *g*, 1 h) and incubated with glutathione-agarose resin (35 μl resin/ml soluble extract) for 30 min. Then, the beads were washed 3 times with 10 volumes of lysis buffer, 3 times with 3 volumes of lysis buffer containing 5 mM ATP and 0.15 mg/ml *E. coli* denatured proteins [35], and finally, 3 times with 10 volumes of P buffer (50 mM Tris–HCl, pH 8.0, 100 mM NaCl, 10 mM MgCl_2_, 10% v/v glycerol). This procedure removes DnaK contamination from TP-containing fusions. For limited proteolysis assays, the fusion proteins were eluted using P buffer + 10 mM glutathione and used immediately.

Recombinant AtClpC2 and AtClpD were expressed and purified as described previously [10,36].

### Protein analysis

Protein samples were subjected to gel electrophoresis in denaturing conditions according to Laemmli [37], using 12% polyacrylamide gels. Samples were also analyzed by Western blotting. Polyacrylamide gels were transferred overnight to nitrocellulose sheets. After transfer, proteins were detected with the corresponding antibody followed by an anti-rabbit alkaline phosphatase-conjugated antibody (GE Healthcare, USA). Detection with the chromogenic substrates 5-bromo-4-chloro-3-indolyl phosphate/nitro blue tetrazolium chloride was carried out as recommended by the suppliers. Band quantification was performed by scanning the blots and by subsequent analysis of the data with GelPro Analyzer software (Media Cybernetics, USA).

The antibodies against GST, FNR and AtClpD were obtained by us. Antibodies against AtClpC2 were purchased from Agrisera AB (Sweden).

### Binding assays

The different fusion proteins were bound to glutathione-agarose beads from cell lysates as described above. Then, 500 μl of the corresponding loaded matrix were added to 500 μl of AtClpC2 or AtClpD in the presence of 5 mM ATP and incubated for 30 min at 25°C. Beads were then pelleted by mild centrifugation and resuspended in 500 μl of P buffer. A 100-μl aliquot was taken from the mixture and centrifugated to collect the resin. The beads were boiled in 50 μl of 2X loading buffer and analyzed by SDS-polyacryamide gels and immunoblotting with antibodies raised against each chaperone. P buffer was added to the remaining resin in an equal volume and the washing procedure was performed as described. This method was repeated three more times. Resin without recombinant proteins or loaded with GST served as controls.

### Limited proteolysis assays

For each assay, 5 μg of the fusion protein GST-TP-FNR were incubated with 2 ng of thermolysin in buffer T (20 mM Tris–HCl, pH 8.0, 400 mM NaCl, 8 mM CaCl_2_). Aliquots corresponding to 1 μg of the fusion protein were taken at 0, 10, 20, 60 and 180 minutes after the addition of the protease. The samples were boiled for 5 minutes with an adequate amount of 2X loading buffer and analyzed by SDS-polyacryamide gels or by immunoblotting using antibodies against GST or FNR.

## Competing interests

The authors declare no competing interests.

## Authors’ contributions

EMB carried out the experiments and helped to draft the manuscript. GLR helped to perform some of the experiments and wrote the manuscript. EAC conceived of the study, its design and coordination and helped to draft the manuscript. All authors read and approved the final manuscript.

## Supplementary Material

Additional file 1: **Table S1.** Sequence detail of the GST-TP-FNR fusion. The table shows the segment of amino acids that separate the GST and FNR cores in the fusion protein Click here for file
