# Supplementary material for: Chloroplastic Hsp100 chaperones ClpC2 and ClpD interact in vitro with a transit peptide only when it is located at the N-terminus of a protein
Source: BMC Plant Biol. 2012 Apr 30;12:57. doi: 10.1186/1471-2229-12-57 (PMC3413601; doi:10.1186/1471-2229-12-57)
Supplement: Additional file 1: — Table S1. Sequence detail of the GST-TP-FNR fusion. The table shows the segment of amino acids that separate the GST and FNR cores in the fusion protein [file 1471-2229-12-57-S1.pdf]

Additional Table 1. Sequence detail of the GST-TP-FNR fusion.

81 amino acids			
----GDHPPK	SDLIEGRGIPR	AAVTA----(TP)----IRA	QVTTEAPAKVVKHS----
1	2	3	4

1- C-terminal exposed amino acids of GST from *S. japonicum*, as observed in the crystal structure [PDB:1UA5].

2- Amino acids sequence from the pGEX-3X cloning site added during the construction of the expression vector.

3- Transit peptide of pea FNR (50 amino acids).

4- N-terminal exposed amino acids of FNR from pea, as observed in the crystal structure [PDB:1QG0].
